# Enhanced metagenomic strategies for elucidating the complexities of gut microbiota: a review

**DOI:** 10.3389/fmicb.2025.1626002

**Published:** 2025-08-26

**Authors:** Xinru Li, Haiyan Lu

**Affiliations:** ^1^Department of Anorectal Surgery, Changsha Hospital of Traditional Chinese Medicine (Changsha Eighth Hospital), Changsha, China; ^2^Department of Anorectal Surgery, The Second Affiliated Hospital of Hunan University of Chinese Medicine, Changsha, China

**Keywords:** gut microbiome, metagenomics, beneficial and harmful microbes, long-read sequencing, multi-omics integration

## Abstract

The human gastrointestinal tract (GIT) is inhabited by a heterogeneous and dynamic microbial community that influences host health at multiple levels both metabolically, immunologically and via neurological pathways. Though the gut microbiota—overwhelmingly *Bacteroidetes* and *Firmicutes*—has essential functions in nutrient metabolism, immune regulation, and resistance to pathogens, its dysbiosis is likewise associated with pathologies, such as inflammatory bowel disease (IBD), obesity, type 2 diabetes (T2D), and neurodegenerative diseases. While conventional metagenomic techniques laid the groundwork for understanding microbial composition, next-generation enhanced metagenomic techniques permit an unprecedented resolution in exploring the functional and spatial complexity of gut communities. Advanced frameworks such as high-throughput sequencing, bioinformatic and multi-omics technologies are expanding the understanding of microbial gene regulation, metagenomic pathways, and host-microbe communication. Beyond taxonomic profiling, they map niche-specific activities of gut microbiota along a dichotomy of facultative mutualism, evidenced by relations of beneficial symbionts, represented here by *Enterobacteriaceae*. In this review, we critically consider the latest approaches (e.g., long-read sequencing, single-cell metagenomics and AI-guided annotation) that mitigate biases stemming from DNA extraction, sequencing depth and functional inference.

## 1 Introduction

The human gastrointestinal tract (GIT) hosts one of the most intricate microbial ecosystems known to science, comprising bacteria, archaea, fungi, and viruses that collectively influence host health through metabolic, immunological, and neurological pathways (Thursby and Juge, 2017). The gut microbiota dominated by the phyla Bacteroidetes and Firmicutes which plays indispensable roles in nutrient metabolism, immune regulation, and pathogen resistance (Thursby and Juge, 2017). These microbial communities ferment dietary fibers into short-chain fatty acids (SCFAs) like butyrate and acetate, which regulate intestinal epithelial integrity and systemic immune responses (Shin et al., 2023). However, disruptions in this delicate balance termed dysbiosis are increasingly linked to pathologies such as IBD, obesity, type 2 diabetes (T2D), and neurodegenerative disorders (Mousa and Ali, 2024). The dualistic nature of gut microbiota, wherein symbionts like *Akkermansia muciniphila* promote metabolic health while pathobionts such as *Enterobacteriaceae* drive inflammation, underscores the need for advanced methodologies to decode microbial dynamics (Mousa and Ali, 2024; Wang et al., 2015).

Traditional metagenomic approaches, pioneered by initiatives like the MetaHIT consortium and the Human Microbiome Project, laid the foundation for cataloging microbial diversity by sequencing 16S rRNA genes and shotgun metagenomes. These efforts revealed over 3.3 million non-redundant genes in the human gut, far exceeding the human genome. Yet, these methods faced limitations: short-read sequencing often fragmented complex genomic regions, while DNA extraction biases skewed taxonomic profiles toward abundant species (Bai et al., 2021). Functional insights remained inferential, relying on homology-based predictions rather than direct measurements of gene expression or metabolic activity (Cozzetto et al., 2016). For instance, early metagenomic studies associated *Faecalibacterium prausnitzii* depletion with IBD but could not clarify whether this reflected causation or correlation (Cozzetto et al., 2016). Similarly, while antibiotic resistance genes (ARGs) were identified in fecal metagenomes, their plasmid-borne mobility and strain-specific distribution required deeper investigation (Xuan et al., 2023).

Emerging enhanced metagenomic strategies now transcend these limitations by integrating high-throughput sequencing, single-cell resolution, and multi-omics frameworks (Forster et al., 2019). Long-read sequencing technologies, such as Oxford Nanopore and PacBio, resolve repetitive genomic elements and structural variations, enabling complete assembly of microbial genomes from complex samples (Forster et al., 2019). This advancement is critical for studying mobile genetic elements like plasmids, which facilitate horizontal gene transfer of ARGs and virulence factors. Complementing this, single-cell metagenomics isolates individual microbial cells, bypassing cultivation biases and revealing genomic blueprints of uncultured taxa (Forster et al., 2019; Tokuda and Shintani, 2024). The Human Gastrointestinal Bacteria Culture Collection (HBC), encompassing 737 whole-genome-sequenced isolates, exemplifies how reference databases enhance taxonomic and functional annotation in metagenomic studies (Mangoma et al., 2024). By mid-2025, such resources have improved subspecies-level classification for nearly 50% of gut microbial sequences, a leap from the 37% genome coverage achieved by earlier projects (Forster et al., 2019; Sugimoto et al., 2019).

These advancements are reshaping translational research. By correlating microbial signatures with clinical outcomes, enhanced metagenomics identifies diagnostic biomarkers and therapeutic targets (Feng et al., 2020). For instance, *Streptococcus anginosus* and *Rothia mucilaginosa* enrichments in HIV-1 patients on antiretroviral therapy correlate with immunodeficiency severity, suggesting microbial markers for treatment monitoring (Forster et al., 2019). Meanwhile, precision editing of gut microbiota through phage therapy or engineered probiotics tailors interventions to individual microbiomes, mitigating adverse drug reactions (Feng et al., 2020).

As the field transitions from observational studies to mechanistic exploration, enhanced metagenomics bridges the gap between microbial taxonomy and host pathophysiology. By resolving strain-level variations, functional pathways, and ecological interactions, these strategies illuminate the gut microbiome’s role in health and disease, paving the way for personalized therapies.

## 2 Gut microbiota: a nexus of health and disease

### 2.1 Gut microbiota in homeostasis

The human gastrointestinal tract harbors a highly diverse and dynamic microbial ecosystem, predominantly composed of over 1,000 bacterial species, with the phyla *Firmicutes* and *Bacteroidetes* being the most dominant (Bull and Plummer, 2014). This microbiota functions as a critical interface between the host and its environment, playing indispensable roles in nutrient metabolism, immune system modulation, and pathogen resistance. Dietary fibers fermented by these microbes generate short-chain fatty acids (SCFAs) notably acetate, propionate, and butyrate—which reinforce intestinal barrier integrity, modulate systemic immune responses, and suppress inflammation by inducing regulatory T-cell differentiation (Yang and Cong, 2021; Rooks and Garrett, 2016).

Beneficial symbionts such as *Faecalibacterium prausnitzii* and *Akkermansia muciniphila* enhance mucosal immunity by producing anti-inflammatory metabolites like indole derivatives and conjugated linoleic acid (Yang and Cong, 2021). Additionally, commensal microbes maintain ecological balance by competitively excluding pathogens and secreting antimicrobial compounds such as bacteriocins, thereby ensuring gastrointestinal homeostasis (Zeng et al., 2016). The complex interplay between specific microbial taxa, their metabolic byproducts, and their roles in health and disease is summarized in Tables 1, 2.

**Table 1:** Role of microorganisms in the health and disease.

Microorganism	Role in the health and disease link	Mechanism	References
*Faecalibacterium prausnitzii*	*Faecalibacterium prausnitzii* produces SCFAs, particularly butyrate, which exert anti-inflammatory effects in IBD by enhancing regulatory T cell differentiation and maintaining intestinal barrier integrity	Butyrate enhances T cell differentiation and strengthens intestinal barrier	Louis et al. (2014)
*Enterobacteriaceae*	Dysbiosis characterized by the overgrowth of *Enterobacteriaceae exacerbates* IBD by promoting inflammation through lipopolysaccharide (LPS)-mediated immune activation	LPS activates TLR4/NF-κB, driving IL-17 and tissue damage	Costa et al. (2020)
*Clostridium scindens*	Development of non-alcoholic fatty liver disease (NAFLD) by producing deoxycholic acid, which inhibits hepatic FXR signaling and promotes lipid accumulation in the liver	Deoxycholic acid inhibits hepatic FXR signaling, promoting steatosis	Arab et al. (2016)
*Akkermansia muciniphila*	*Akkermansia muciniphila* contributes to the improvement of metabolic syndrome by degrading mucin, thereby strengthening the gut barrier and enhancing insulin sensitivity	Mucin degradation enhances gut barrier function and insulin sensitivity	Vogt et al. (2018) and Plovier et al. (2016)
*Bacteroides fragilis*	Gut microbiota-linked colorectal cancer (CRC) refers to the development or progression of colorectal cancer influenced by specific microbial species	Polysaccharide A activates Wnt/β-catenin signaling in epithelial cells	Dejea et al. (2018)
*Lactobacillus rhamnosus*	Reduction in anxiety- and depression-like behaviors	GABA synthesis and vagal nerve stimulation regulate serotonin availability	Bravo et al. (2011)
*Bifidobacterium longum*	Alleviation of symptoms in irritable bowel syndrome (IBS)	Competitive exclusion of pathogens and reinforcement of mucosal defenses	Chen et al. (2023)
*Bacteroidetes*-enriched communities	Fecal microbiota transplantation-induced remission of *C. difficile* infection	Restores bile acid metabolism and niche competition against pathogens	van Nood et al. (2013)

**Table 2:** Microbial metabolites and their role in the human health and disease.

Metabolites	Role in health and disease	Mechanism	References
Trimethylamine N-oxide (TMAO)	Neuroinflammation and play important role in Alzheimer’s disease	TMAO crosses BBB, triggers microglial activation, and promotes Aβ aggregation	Vogt et al. (2018)
Resistant starch fermentation	Diet-microbiota crosstalk and improved glycemic control in T2D	Acetate stimulates GPR43, enhancing insulin secretion and β-cell function	Chen et al. (2024)

### 2.2 Dysbiosis and metabolic-immune dysfunction

Disruptions in microbial equilibrium, termed dysbiosis, can be triggered by factors like high-sugar diets, antibiotic overuse, and reduced fiber intake. Dysbiosis favors pro-inflammatory taxa such as *Enterobacteriaceae* and *Fusobacterium nucleatum*, while depleting protective microbes (Forster et al., 2019; Zeng et al., 2016). These alterations impair the intestinal barrier, allowing microbial products like lipopolysaccharide (LPS) and flagellin to translocate into systemic circulation. Such pathogen-associated molecular patterns (PAMPs) activate Toll-like receptors (TLRs) and NOD-like receptors (NLRs), triggering NF-κB signaling and systemic low-grade inflammation that underlies metabolic diseases such as obesity and T2D (Potrykus et al., 2021).

In IBD for instance, blooms of *Enterobacteriaceae* are associated with elevated IL-17 production and mucosal damage (Zeng et al., 2016). Similarly, in colorectal cancer (CRC), overabundance of *Bacteroides fragilis* promotes oncogenic Wnt/β-catenin signaling through polysaccharide A, emphasizing how pathobionts exploit dysbiosis to drive disease progression (Bull and Plummer, 2014; de Vos et al., 2022).

### 2.3 Systemic impacts: gut-liver, gut-joint, and gut-brain axes

The impact of gut dysbiosis extends to extraintestinal sites via multiple host-microbe interaction axes.

#### 2.3.1 Gut-liver axis

The gut-liver axis represents a critical communication network influenced by gut microbiota and their metabolites. Altered microbial composition, particularly the enrichment of *Clostridium scindens*, leads to increased production of secondary bile acids such as deoxycholic acid, which disrupts farnesoid X receptor (FXR) signaling in the liver. Impaired FXR activity affects lipid and glucose metabolism, bile acid homeostasis, and promotes hepatic inflammation and steatosis. These disruptions contribute significantly to the onset and progression of NAFLD. Moreover, gut-derived endotoxins entering the portal circulation exacerbate liver injury by activating inflammatory pathways and fibrogenesis (Hrncir, 2022).

#### 2.3.2 Gut-joint axis

The gut-joint axis highlights the interplay between gut microbiota and autoimmune joint diseases. In rheumatoid arthritis (RA), an increased abundance of *Prevotella copri* promotes T-helper 17 (Th17) cell differentiation, leading to heightened systemic inflammation and joint destruction. These immune alterations are driven by microbial antigens that trigger proinflammatory cytokine release, including IL-17 and TNF-α. Similar gut-immune interactions are observed in systemic lupus erythematosus (SLE), where dysbiosis alters mucosal tolerance and promotes autoantibody production. These findings suggest that gut microbiota play a pivotal role in initiating and perpetuating autoimmune responses, making the gut a potential therapeutic target in RA and SLE (Yang and Cong, 2021).

#### 2.3.3 Gut-brain axis

The gut-brain axis represents a bidirectional communication network between the gut microbiota and the central nervous system, mediated through immune, neuroendocrine, and metabolic pathways. Dysbiosis can reduce the availability of serotonin precursors and impair γ-aminobutyric acid (GABA) synthesis, contributing to anxiety and depression. Moreover, microbial metabolites such as TMAO and diminished short-chain fatty acids (SCFAs) exacerbate neuroinflammation and compromise blood-brain barrier integrity, factors implicated in Alzheimer’s disease. These disruptions highlight the critical role of gut microbiota in maintaining neurological health and suggest that microbial modulation may offer therapeutic avenues for various neuropsychiatric disorders (Yang and Cong, 2021).

### 2.4 Cardiovascular and multisystemic consequences

Beyond metabolic and neurological impacts, gut microbial metabolites significantly influence cardiovascular health and multisystemic processes. One such metabolite, phenylacetylglutamine (PAGln), produced by gut microbes from dietary phenylalanine, has been shown to enhance platelet reactivity, thereby increasing the risk of atherosclerosis and thrombotic events. Additionally, metabolites like TMAO contribute to endothelial dysfunction, inflammation, and lipid metabolism disturbances. These microbial products not only affect cardiovascular physiology but also have systemic effects, linking gut dysbiosis to broader health issues, including chronic kidney disease and metabolic syndrome, underscoring the gut microbiota’s central role in maintaining systemic homeostasis and disease susceptibility (Rooks and Garrett, 2016).

## 3 Evolution of metagenomic methodologies

**Figure 1:**
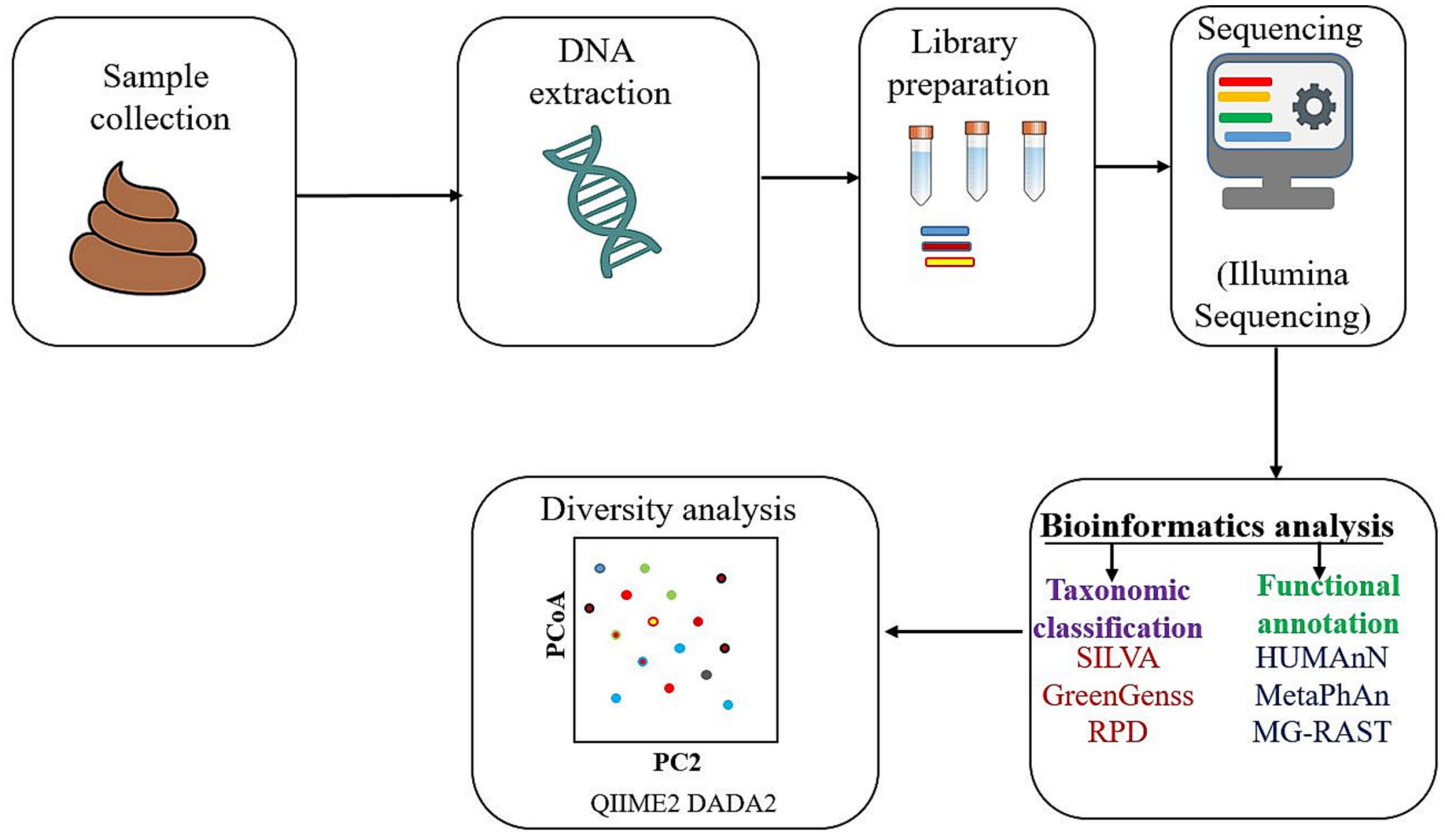
An overview of a metagenomic method for analysing the gut microbiome.

Before investigating the functional involvement of microbial communities in disease pathophysiology, it is essential to accurately identify and characterize these communities with high sensitivity and specificity. Metagenomics enables such identification through two major culture-independent approaches: 16S rRNA gene amplicon sequencing and shotgun metagenomic sequencing (Jovel et al., 2016; Ranjan et al., 2016; Ghaisas et al., 2016; Bastiaanssen et al., 2018; Bastiaanssen et al., 2018). The 16S rRNA approach is mainly used for taxonomic profiling by amplifying conserved bacterial gene regions, while shotgun metagenomics provides comprehensive insights into both taxonomic composition and functional potential by sequencing total genomic DNA from a sample (Bastiaanssen et al., 2018) (Figure 1).

The analysis workflow generally begins with sample collection, DNA extraction, and library preparation. Sequencing is typically performed on high-throughput platforms such as Illumina due to its greater efficiency and accuracy. The resulting DNA sequences are then analyzed using bioinformatics pipelines. Taxonomic classification is performed by comparing sequence reads to curated databases such as SILVA, GreenGenes, or RDP. Functional annotations are obtained using tools like HUMAnN, MetaPhlAn, or MG-RAST, which map sequences to gene and pathway databases (Reck et al., 2015). To evaluate differences in microbial composition across conditions or cohorts, diversity metrics such as alpha (within-sample) and beta diversity (between-sample) are calculated. Tools like QIIME2 and DADA2 enable comprehensive statistical analyses, including principal coordinate analysis (PCoA) and differential abundance testing (Reck et al., 2015; Balvočiūtė and Huson, 2017).

### 3.1 16S rRNA gene sequencing

The 16S rRNA gene is a highly conserved and universally present genetic marker in bacterial and archaeal genomes, making it one of the most widely used tools for microbial taxonomic profiling (Bukin et al., 2019). Cryan et al. (2019) highlighted that 16S rRNA sequencing is a cost-effective, widely accessible method for studying microbial communities, particularly in the gut microbiome of humans, mice, and insects. This method allows for the assessment of microbial diversity and relative abundance using next-generation sequencing technologies.

In this approach, PCR is used to amplify conserved regions of the 16S rRNA gene, and the variable regions are sequenced to distinguish different taxa. The resulting sequences, or amplicons, are grouped into operational taxonomic units (OTUs), typically at 97% sequence similarity (Bukin et al., 2019). Alternatively, amplicon sequence variants (ASVs), generated through denoising algorithms such as DADA2 or Deblur, offer higher resolution and reduced error rates (Prodan et al., 2020).

However, while 16S rRNA is the most commonly used genetic marker for bacterial identification, it is not without limitations. Critically, the 16S rRNA gene is not always present as a single copy within bacterial genomes-some bacteria carry multiple copies with varying sequences. This gene copy number variability can distort estimates of microbial abundance and skew taxonomic profiles, especially when comparing species with different 16S copy numbers. Moreover, the resolution of 16S rRNA sequencing is generally limited to the genus level, often lacking the specificity to accurately identify species (Knight et al., 2018; Callahan et al., 2017).

Different strategies for OTU clustering (e.g., *de novo*, closed-reference) further influence the accuracy and comparability of results (Callahan et al., 2017). Despite these challenges, 16S rRNA sequencing remains a widely adopted approach for large-scale microbiome studies, especially when focusing on bacterial and archaeal populations across various health and disease conditions (Prodan et al., 2020).

### 3.2 Shotgun metagenomic sequencing

Shotgun metagenomic sequencing provides a comprehensive and accurate depiction of microbial communities by sequencing all DNA present in a sample, surpassing 16S rRNA in species-level resolution and functional potential (Dovrolis et al., 2017; Ranjan et al., 2016). However, this approach is expensive, data-intensive, and technically complex. Crucially, the accuracy and reproducibility of results heavily depend on sample handling, conservation, and preparation procedures-critical elements often overlooked. Temperature control, cell-size filtration, and DNA extraction methods directly influence microbial representation and community structure, introducing variability across studies.

Inconsistent sample processing, such as improper freezing or delayed processing, may degrade DNA, affect microbial viability, or bias detection of certain taxa. Moreover, variations in DNA extraction protocols can result in differential lysis efficiency, leading to underrepresentation of key microbial groups, particularly Gram-positive species. Filtration by cell size, if improperly performed, may skew microbial diversity by excluding small-sized microbes or including host DNA (Ranjan et al., 2016).

**Figure 2:**
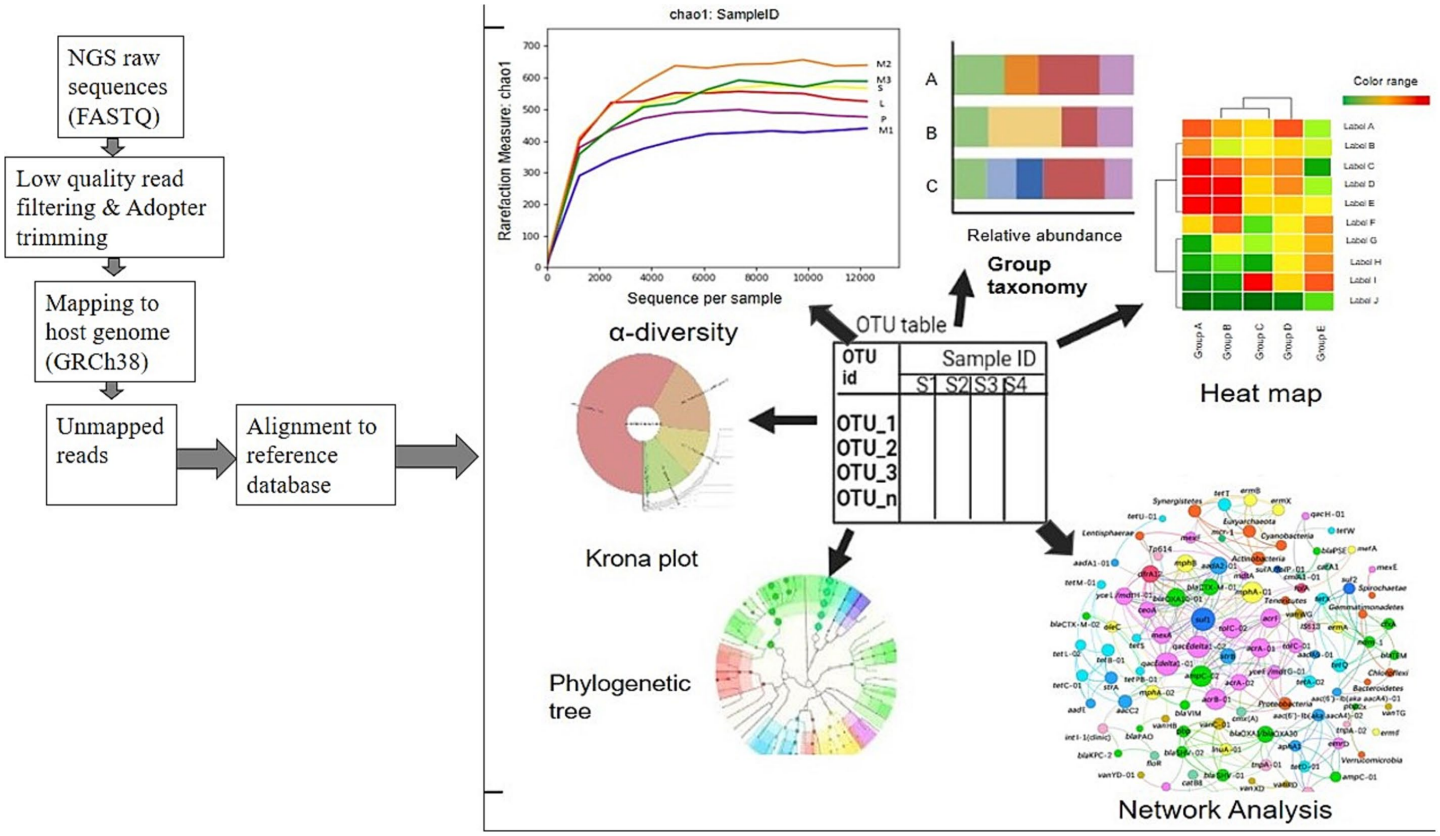
An overview of whole metagenome analysis of gut microbiota analysis.

The NGS library construction for RNA or DNA uses a procedural methodology that results in variations between studies. Regardless of whether a complete sample is being sequenced, this methodology entails the generation of infinitesimal reads ranging from 25 to 500 base pairs. This allows the identification of microorganisms that are either unknown or exist in minute quantities. Chiu and Miller (2019) reported that extensive bioinformatics preprocessing tools are required, including pruning, merging, assembly, scaffolding, and mapping tools. Following the sequencing procedure, distinct sequences of the microbial components of the samples will be produced in fasta or fastq files, along with a mapping file that contains all the necessary metadata associated with the sample. It is said that these files serve as inputs to the subsequent identification of the species to which the sequences belong and the assignment of taxonomy to the sequences (Caporaso et al., 2010). Using the term OTU as shown in Figure 2, it is possible to identify groups of similar sequences that have the potential to represent a distinct taxonomic classification based on these similarities (Caporaso et al., 2010).

Although this method is not without faults, it is remarkably effective for clustering sequences with 97% similarity. Using phylogenetic alignment, one sequence is selected per OTU to represent its corresponding taxa. Numerous bioinformatics techniques and algorithms have been devised in the field of shotgun and 16S rRNA metagenomics, either as independent homology- and prediction-based methods or as components of more comprehensive workflows (Singh et al., 2022; Edgar, 2018).

Multiple investigations, utilising 16S rRNA analysis, have demonstrated a connection between the gut microbiota and general well-being (Kinross et al., 2011; Schippa and Conte, 2014; Ganesan et al., 2018). An extensive examination of the gut metagenome, including WGS, can enhance our understanding of the development of illnesses and allow for the discovery of new therapeutic targets. This occurs because there is a chance of discovering small genetic differences among species that cause changes in physical traits, ultimately resulting in the development of diseases. For instance, WGS investigations carried out with *Citrobacter* spp. have shown that genetic differences within the species lead to changes in their observable characteristics and ability to adapt to different environments (Karlsson et al., 2013).

At present, the use of Illumina shotgun sequencing of stool samples is widely prevalent in the field of whole genome WGS studies of the gut microbiome. This is primarily because the gut harbours a multitude of diverse microbial species, thereby necessitating a thorough sequencing process with a coverage of at least 20 times. The purpose of such extensive sequencing is to investigate and analyze individual communities within the gut microbiome that possess low abundance (Karlsson et al., 2013). However, it is important to analyse the substantial amount of WGS data, particularly in the form of short reads, which poses significant challenges. This is needful because the gut microbiome is home to a wide range of bacterial species, ranging from hundreds to thousands, all of which exhibit varying levels of abundance. Complicating matters further, there is a lack of taxonomic identification available for the majority of these species, further exacerbating the analytical complexities (Karlsson et al., 2013; Caporaso et al., 2010).

### 3.3 Bioinformatics pipelines for gut microbial analysis

Bioinformatics pipelines are essential for analyzing gut microbiota, allowing researchers to process and interpret complex metagenomic datasets efficiently. They support taxonomic, functional, and strain-level analyses, providing insights into microbial diversity and health associations. Table 3 highlights key, updated bioinformatics tools commonly used in gut microbiota studies for comprehensive and accurate data analysis.

**Table 3:** Bioinformatics pipelines for gut microbiota analysis.

Tools	Main function	Advantage	Platform	References
mothur	Microbial ecology analysis of gut microbiota	This tool has the ability of fast process of large sequence data set	Linux, macOS, Windows	Schloss et al. (2009)
MEGAN	Taxonomic analysis and visualization	This tool is applicable for the analysis of large metagenomic shotgun sequencing data	Windows, macOS	Huson et al. (2011)
MetaPhinder	Identification of phages in metagenomic data	Phage identification in metagenomic datasets	Linux	Namiki et al. (2012)
MOCAT	Metagenomic data analysis	This tool has important application in the generation and assembly of taxonomic profiles and assemble metagenomes	Linux	Kultima et al. (2012)
QIIME	Microbiome analysis pipeline	QIIME is use for Net-work analysis of meta-genomic data and histograms between microbial sample diversity or histrograms within the sample	Linux, macOS, Windows	Caporaso et al. (2010) and Li (2009)
MetaPhlAn	Microbial composition profiling	This computational tool is applicable for the faster profiling of the microbial composition of microbial communities by clade-specific marker genes	Linux, macOS	Segata et al. (2012)
ConStrains	Strain-level analysis of metagenomic data	High-resolution strain identification from metagenomes	Linux	Luo et al. (2015)
MEBS	Metagenomic data processing and analysis	Efficient analysis of metagenomic sequencing data	Linux	Tarracchini et al. (2023)
Kraken2/Bracken	Taxonomic classification from shotgun data	Ultrafast and highly accurate with abundance estimation (Bracken)	Linux	Lu et al. (2024)
HUMAnN 3	Functional profiling of metagenomes	Improved accuracy in pathway reconstruction and gene-family abundance	Linux	Beghini et al. (2021)
StrainPhlAn 4	Strain-level resolution of metagenomic data	Accurate strain tracking based on MetaPhlAn markers	Linux	Blanco-Míguez et al. (2023)

These pipelines streamline data processing from raw sequences to biological insights, enabling researchers to study gut microbiota’s role in health and disease. By integrating these tools, researchers can uncover novel therapeutic targets and biomarkers, advancing our understanding of microbiome-disease interactions.

## 4 Enhanced metagenomic strategies: overcoming biases and gaps

Enhanced metagenomic strategies address limitations in traditional methods by integrating advanced technologies. Long-read sequencing resolves genomic complexities, while single-cell metagenomics bypasses culturability biases. AI-driven annotation improves functional inference, overcoming biases in DNA extraction and sequencing depth (Forbes et al., 2017). These advancements enable precise characterization of microbial communities, revealing strain-level variations and niche-specific activities. By transcending taxonomic profiling, they illuminate the dualistic nature of gut microbiota, distinguishing beneficial symbionts from pathobionts. These strategies unlock microbial roles in health and disease, offering biomarkers for diagnostics and therapeutic targets (Yan et al., 2020).

### 4.1 Long-read sequencing for genomic resolution and structural variation analysis

Long-read sequencing technologies, such as Oxford Nanopore and PacBio SMRT, have transformed metagenomic analyses by enabling the resolution of complex genomic regions and structural variations that evade detection by short-read methods. These platforms generate reads spanning thousands of base pairs, allowing for the assembly of complete microbial genomes, including repetitive elements, plasmids, and mobile genetic elements critical for antibiotic resistance and virulence (Panahi et al., 2024; Satam et al., 2023). Unlike short-read sequencing, which fragments these regions, long-read data preserves genomic context, enhancing functional annotation accuracy and strain-level resolution. For instance, long-read sequencing has resolved strain-specific adaptations in *Citrobacter* spp., linking genetic variations to phenotypic changes in environmental adaptability and pathogenicity (Logsdon et al., 2020). This capability is vital for studying horizontal gene transfer dynamics in dysbiotic gut communities, where plasmid-borne resistance genes proliferate. Additionally, long-read sequencing mitigates biases in taxonomic profiling by capturing low-abundance taxa and uncultured species, which are often underrepresented in traditional workflows (Ruscheweyh et al., 2022). Long-read sequencing technologies overcome limitations of short-read methods by resolving repetitive genomic regions, structural variations, and microbial mobile elements. Below is a comparative analysis of key platforms and their applications in gut microbiota research (Table 4).

**Table 4:** Comparative analysis of key platforms and their applications in gut microbiota.

Sequencing platform	Read length	Error rate	Advantages	Applications in gut microbiota	References
Oxford Nanopore	Up to 2 Mb	~5–10% (recent <5% with duplex reads)	Real-time sequencing, detects plasmids	Structural variation analysis, mobile elements, antibiotic resistance profiling	Cantalapiedra et al. (2021)
PacBio SMRT	10–25 kb (CLR)	~10–13% (CLR), <1% (HiFi)	High accuracy, complete genome assembly	Strain-level resolution, functional pathway annotation	Ma et al. (2024)
PacBio HiFi	15–25 kb	<0.1%	Ultra-high accuracy, hybrid assemblies	Precision metagenomics, rare taxa and variant detection	Salmaso et al. (2022)
Illumina Short-Read	100–300 bp	<0.1%	High accuracy, cost-effective, high throughput	16S rRNA profiling, taxonomic/functional profiling and diversity analysis	Ma et al. (2024) and DSouza et al. (2020)

The application of long-read sequencing in gut microbiota research has unveiled niche-specific microbial activities and structural variations driving disease. For example, it has identified mucosal-associated *Escherichia coli* strains in IBD patients with intact virulence operons, correlating with NF-κB-mediated inflammation (Schirmer et al., 2019). Similarly, long-read assemblies of *Akkermansia muciniphila* genomes have revealed strain-specific mucin degradation pathways critical for metabolic health (Ouwerkerk et al., 2022). Despite its advantages, challenges persist, including higher costs, computational demands for data processing, and the need for high-quality DNA input. Platforms like PacBio HiFi address error rate limitations, offering >99% accuracy for clinical-grade analyses (Oehler et al., 2023). As these technologies mature, they will bridge gaps in functional and structural metagenomics, enabling precision interventions targeting microbial contributions to diseases like T2D and colorectal cancer (Oehler et al., 2023).

### 4.2 Single-cell metagenomics: decoding uncultured taxa and strain heterogeneity

Single-cell metagenomics has emerged as a transformative strategy to resolve uncultured microbial taxa and strain-level heterogeneity, overcoming limitations of bulk sequencing methods that obscure rare or low-abundance species (Xu and Zhao, 2018). By isolating individual microbial cells via microfluidics or fluorescence-activated cell sorting, this approach bypasses PCR amplification biases and culturability challenges, enabling direct sequencing of genomes from “microbial dark matter.” For example, single-cell genomics has expanded the Human Gastrointestinal Bacteria Culture Collection (HBC), providing genomic blueprints for novel species like *Saccharimonadia* and uncultured *Clostridiales*, which evade traditional cultivation (Yu et al., 2022). This technique resolves strain-specific genetic variations, such as antibiotic resistance gene clusters in *Escherichia coli* subpopulations or mucin-degrading adaptations in *Akkermansia muciniphila* strains, critical for understanding niche-specific functionalities (Ouwerkerk et al., 2022). Coupled with AI-driven annotation pipelines, single-cell data enhances reference databases, improving taxonomic classification accuracy by 30% compared to conventional methods (Erfanian et al., 2023). Furthermore, it elucidates horizontal gene transfer dynamics, revealing plasmid-mediated virulence factor exchange in dysbiotic gut communities. By decoding strain-specific metabolic capabilities and host-interaction genes, single-cell metagenomics bridges gaps in functional annotation, offering insights into microbial contributions to diseases like IBD and T2D, while guiding precision probiotics and phage therapies (Ott and Mellata, 2022).

### 4.3 Integrative multi-omics frameworks: bridging genomics, transcriptomics, and metabolomics

Integrative multi-omics frameworks synergize genomic, transcriptomic, and metabolomic datasets to unravel the functional and spatial dynamics of gut microbiota. By coupling shotgun metagenomics with metatranscriptomics, researchers can map microbial gene expression to metabolic pathways, revealing how taxa like *Akkermansia muciniphila* modulate mucin degradation or *Faecalibacterium prausnitzii* regulate butyrate synthesis in health and disease (Chetty and Blekhman, 2024). For instance, metatranscriptomic profiling in T2D patients identified upregulated carbohydrate metabolism genes in *Muribaculaceae*, linking microbial activity to glycemic dysregulation (Zhu and Goodarzi, 2020). Tools like HUMAnN3 quantify pathway contributions across taxa, while KEGG and EggNOG databases annotate gene functions, enabling systems-level insights into host-microbe crosstalk (Hernández-Plaza et al., 2022). These frameworks resolve strain-specific adaptations, such as *Citrobacter* spp. genetic variations influencing environmental adaptability, and track horizontal gene transfer of antibiotic resistance genes via plasmids. There are few important integrative multi-omics methods for gut microbiota analysis are listed in Table 5.

**Table 5:** Integrative multi-omics approach: application and mechanism.

Framework component	Application	Mechanism	Example	References
Shotgun metagenomics	Species- and strain-level microbial identification	High-throughput sequencing of all microbial DNA in a sample, enabling functional pathway annotation	Identified *Citrobacter* spp. strain variations linked to environmental adaptability	Gallegos et al. (2020) and Sequeira et al. (2022)
Metatranscriptomics	Gene expression profiling of active microbial pathways	RNA sequencing to map microbial transcripts, linking gene activity to metabolic outputs	Upregulated carbohydrate metabolism genes in *Muribaculaceae* in T2D patients	Ojala et al. (2023) and Vaccaro et al. (2024)
Multi-omics platforms (HUMAnN3)	Integrating genomic, transcriptomic, and metabolomic data streams	Hierarchical alignment of reads to KEGG/MetaCyc pathways, quantifying taxonomic contributions	Mapped *Akkermansia muciniphila* mucin degradation pathways to host metabolic health	Sequeira et al. (2022) and Vaccaro et al. (2024)
Metabolomics integration	Linking microbial activity to host physiology	NMR/LC-MS detects metabolites (e.g., SCFAs, TMAO) correlated with microbial gene expression	Reduced butyrate and elevated LPS in T2D linked to *Bifidobacterium* depletion	Gallegos et al. (2020) and Tsouka and Masoodi (2023)
AI-driven annotation (MetaBGC)	Functional pathway prediction and biosynthetic gene cluster identification	Machine learning models predict gene clusters from metagenomic reads	Identified type II polyketide BGCs in gut microbiota with antimicrobial properties	Vaccaro et al. (2024) and Tsouka and Masoodi (2023)

Metabolomic integration further contextualizes microbial activity by identifying metabolites like SCFAs or TMAO that mediate host physiology. For example, NMR-based metabolomics paired with metagenomics revealed reduced butyrate and elevated LPS in T2D, correlating with *Bifidobacterium* depletion and *Enterobacteriaceae* blooms (Zou et al., 2022). Multi-omics platforms like MetaboAnalyst and XCMS align metabolite profiles with microbial gene expression, clarifying mechanisms like bile acid transformations by *Clostridium scindens* in non-alcoholic fatty liver disease (Eicher et al., 2020). However, challenges persist in data harmonization, as batch effects and platform-specific biases require advanced normalization algorithms (Adamer et al., 2022). Emerging AI-driven pipelines, such as MetaBGC, predict biosynthetic gene clusters from metagenomic reads, accelerating therapeutic discovery (Sahayasheela et al., 2022). By bridging omics layers, these frameworks decode microbial contributions to disease, paving the way for precision probiotics and microbiome-editing therapies (Sahayasheela et al., 2022).

### 4.4 Artificial intelligence and machine learning in functional annotation and pathway prediction

The integration of artificial intelligence (AI) and machine learning (ML) into metagenomics has transformed functional annotation and pathway prediction, addressing limitations of traditional homology-based methods such as database bias and incomplete reference genomes. AI-driven tools like MetaBGC leverage probabilistic models to identify biosynthetic gene clusters (BGCs) directly from metagenomic sreads, enabling the discovery of geographically stratified metabolites with therapeutic potential, such as type II polyketides with antimicrobial properties (Vaccaro et al., 2024; Tsouka and Masoodi, 2023). For instance, MetaBGC identified BGCs in gut microbiota linked to dietary adaptations, offering insights into evolutionary strategies of uncultured taxa like Clostridiales (Vaccaro et al., 2024). Similarly, DeepARG, a deep learning framework, predicts ARGs from raw sequencing data with 20% higher accuracy than BLAST-based methods, critical for tracking plasmid-borne resistance genes in dysbiotic communities (Tsouka and Masoodi, 2023; Quainoo et al., 2017). These models bypass reliance on reference databases, enabling annotation of “microbial dark matter” that lacks representation in public repositories (Kanehisa et al., 2015).

ML frameworks also enhance pathway prediction by integrating multi-omics data. HUMAnN3 employs hierarchical alignment to KEGG and MetaCyc databases, quantifying taxonomic contributions to metabolic pathways. For example, it mapped *Akkermansia muciniphila* mucin degradation pathways to improved insulin sensitivity in metabolic syndrome (Vaccaro et al., 2024; Kanehisa et al., 2015). AI models trained on metatranscriptomic data have linked upregulated carbohydrate metabolism genes in *Muribaculaceae* to glycemic dysregulation in T2D (Shen et al., 2015). Tools like eggNOG-mapper use DIAMOND aligners to assign orthologous groups, improving functional annotation of genes from fragmented metagenomic assemblies (Tsouka and Masoodi, 2023). Meanwhile, MMvec, a neural network, predicts microbe-metabolite interactions, such as *Faecalibacterium prausnitzii*-derived butyrate synthesis correlating with IBD remission (Vaccaro et al., 2024; Shen et al., 2015). The key AI/ML tools for functional annotation and pathways prediction are listed in the Table 6.

**Table 6:** AI/ML tools for functional annotation and pathway prediction.

Tool	Function	Mechanism	Application example	References
MetaBGC	BGC identification	Probabilistic model detects biosynthetic pathways from metagenomic reads	Type II polyketide discovery in gut microbiota	Vaccaro et al. (2024) and Tsouka and Masoodi (2023)
DeepARG	Antibiotic resistance prediction	Deep neural network classifies ARGs from sequencing data	Plasmid-borne β-lactamase detection in *E. coli*	Tsouka and Masoodi (2023) and Quainoo et al. (2017)
HUMAnN3	Pathway quantification	Hierarchical alignment to KEGG/MetaCyc databases	*A. muciniphila* mucin degradation pathway mapping	Vaccaro et al. (2024) and Kanehisa et al. (2015)
eggNOG-mapper	Orthologous group assignment	DIAMOND aligner matches sequences to evolutionary gene clusters	Functional annotation of uncultured *Clostridiales*	Tsouka and Masoodi (2023) and Huerta-Cepas et al. (2017)
MMvec	Microbial metabolite interaction prediction	Neural network models microbe-metabolite covariation	Linking *Faecalibacterium* butyrate synthesis to IBD remission	Vaccaro et al. (2024) and Huerta-Cepas et al. (2017)

AI/ML frameworks bridge gaps in strain-specific gene function and host-microbe crosstalk. For example, *Citrobacter* spp. strain variations influencing environmental adaptability were resolved using PacBio HiFi sequencing and ML-driven annotation (Quainoo et al., 2017). These tools are pivotal for identifying therapeutic targets, such as *Clostridium scindens*-mediated bile acid metabolism in NAFLD (Kanehisa et al., 2015). As the field advances, AI-driven metagenomics will underpin precision probiotics and microbiome-editing therapies, translating microbial ecology into clinical innovation (Vaccaro et al., 2024; Huerta-Cepas et al., 2017).

## 5 Conclusion

Enhanced metagenomic strategies have revolutionized our understanding of gut microbiota by transcending the limitations of traditional approaches. Long-read sequencing resolves structural variations and plasmids, enabling complete genome assemblies of uncultured taxa and strain-level insights into pathobionts like *Citrobacter* spp. Single-cell metagenomics deciphers “microbial dark matter,” while AI-driven tools (MetaBGC, HUMAnN3) predict biosynthetic pathways and antibiotic resistance genes with unprecedented accuracy. Multi-omics frameworks integrate genomic, transcriptomic, and metabolomic data, linking microbial activity to host phenotypes-such as *Akkermansia muciniphila’s* mucin degradation in metabolic health or *Muribaculaceae’s* upregulated carbohydrate metabolism in T2D. These strategies identify biomarkers (e.g., butyrate-producing *Bifidobacterium* depletion in T2D) and therapeutic targets, such as *Clostridium scindens*-mediated bile acid metabolism in NAFLD. Despite challenges in standardization and computational demands, enhanced metagenomics bridges observational and mechanistic research, paving the way for precision probiotics, microbiota identification and microbiome-editing interventions. As the field advances, these tools will be pivotal in translating microbial ecology into actionable clinical strategies, transforming our approach to managing chronic diseases.
